# The TLR-2/TonEBP signaling pathway regulates 29-kDa fibronectin fragment-dependent expression of matrix metalloproteinases

**DOI:** 10.1038/s41598-021-87813-8

**Published:** 2021-04-26

**Authors:** Hyun Sook Hwang, Mi Hyun Lee, Hyun Ah Kim

**Affiliations:** 1 Division of Rheumatology, Department of Internal Medicine, Hallym University Sacred Heart Hospital, 896, Pyungchon, Anyang, Gyeonggi 14068 Korea; 2 Institute for Skeletal Aging, Hallym University, Chuncheon, 24251 Korea

**Keywords:** Osteoarthritis, Cartilage, Mechanisms of disease

## Abstract

Tonicity-responsive enhancer-binding protein (TonEBP; nuclear factor of activated T cells 5) is a transcription factor that responds to changes in osmolality. However, recent studies have shown that it also modulates immune responses under inflammatory conditions independently of hyperosmolality. Fibronectin fragments (FN-fs), which are abundant in the synovial fluid of patients with osteoarthritis (OA), induce expression of matrix metalloproteinases (MMPs) via the toll-like receptor-2 (TLR-2) signaling pathway. In this study we examined whether TonEBP is involved in 29-kDa FN-f-induced expression of MMPs. The expression of TonEBP was significantly higher in human osteoarthritis compared with normal cartilage samples. 29-kDa FN-f affected the expression of MMPs 1, 3, and 13 via TonEBP, and expression and nuclear accumulation of TonEBP were induced by activation of the phospholipase C/NF-κB/MAPK signaling pathway and, in particular, modulated by TLR-2. In addition, 29-kDa FN-f induced the expression of osmoregulatory genes, including *Tau-T, SMIT, *and* AR*, as well as voltage-dependent calcium channels via the TonEBP/TLR-2 signaling pathway. These results show that 29-kDa FN-f upregulates MMPs in chondrocytes via the TLR-2/TonEBP signaling pathway.

## Introduction

The hallmark of osteoarthritis (OA) is destruction of the articular cartilage, which results from the disturbance of the equilibrium between the anabolic and catabolic activities of chondrocytes and loss of the extracellular matrix (ECM)^1^. Due to the high concentration of negatively charged sulfated glycosaminoglycans on aggrecan (ACAN), cartilage tends to attract sodium ions and water within the tissue, resulting in elevated osmolarity and high osmotic pressure compared with surrounding tissues, allowing it to resist compressive loads in the joint^2^. During the progression of OA, reduced proteoglycans and loss of matrix integrity affect osmolarity as well as water content, which compromise its capacity to withstand mechanical stress.

Tonicity-responsive enhancer-binding protein (TonEBP) is a crucial transcription factor required for the regulation of cellular homeostasis in response to osmotic stress^3,4^. TonEBP was identified as a protein specifically interacting with the regulatory sequence element tonicity-responsive enhancer, located within a 13-bp segment of the promoter region of the sodium chloride/betaine cotransporter gene, in renal medullary cells^5^. TonEBP is also known as nuclear factor of activated T cells 5 (NFAT5) because it shares 43% sequence homology with NFATs 1–4, which play pivotal roles in the transcription of cytokine genes and other genes critical for the immune response. Unlike monomeric members of the NFAT family, however, TonEBP exists as a homodimer^6,7^. In a previous study, suppression of TonEBP led to significant downregulation of ACAN promoter activity in nucleus pulposus (NP) cells of the intervertebral disc^8^. Another study showed that β1,3-glucuronosyltransferase (GlcAT-I), an enzyme required for the synthesis of chondroitin sulfate chains, was positively regulated by TonEBP in response to ionophores in intervertebral disc cells^9^. These findings indicate that TonEBP upregulates ECM in response to changes in osmolarity in the intervertebral disc cartilage, which acts as a shock absorber of the spine.

However, it was also reported that TonEBP expression is induced in response to inflammatory stimuli, including interferon gamma and interleukin (IL)-4 in immune cells, independently of hyperosmolarity^10^. TonEBP is highly expressed in rheumatoid synovium, and its activity is increased by proinflammatory cytokines, such as IL-1β and tumor necrosis factor-α (TNF-α)^11^. Mice with TonEBP haploinsufficiency (TonEBP^+/−^) developed a very limited degree of synovial proliferation, as well as decreased angiogenesis, and exhibited nearly complete suppression of experimentally induced arthritis^11^. In NP chondrocytes, RNA sequencing showed that 8.5% of TNF-α transcriptional responses were TonEBP dependent, and these genes were enriched in pathways related to inflammatory responses and inhibition of matrix metalloproteinases (MMPs)^12^. These results suggest that tonicity-independent stimulation of TonEBP plays a pleiotropic role in inflammation, angiogenesis, cell proliferation, and cartilage degeneration. However, the influence of TonEBP on OA articular cartilage has not been reported.

Previously, we reported that chondrocytes upregulate catabolic mediators in response to 29-kDa fibronectin fragment (FN-f), a degradation product of fibronectin, and perpetuate matrix damage via toll-like receptor (TLR)-2^13–15^. The objective of this study was to determine whether TonEBP is activated in OA by 29-kDa FN-f and to identify the signaling mechanisms involved. The influence of TonEBP on the catabolic response mediated by 29-kDa FN-f and the cross-regulation of TLR-2 and TonEBP was examined as well.

## Experimental methods

### Materials

29-kDa amino-terminal fibronectin fragment (29-kDa FN-f) from human plasma, U73122 (a phosphoinositide-specific phospholipase C (PLC)-γ-specific inhibitor), SP600125 (c-Jun N-terminal kinase (JNK) inhibitor), PD98059 (extracellular signal-regulated kinase (ERK) inhibitor), SB203580 (p38 inhibitor), Bay 11-7082 (nuclear factor kappa-light-chain-enhancer of activated B cells (NF-κB) inhibitor), 2‐bis(o‐aminophenoxy)ethane‐N,N,N′,N′‐tetraacetic acid acetoxymethyl ester (BAPTA, a permeable Ca^2+^ chelator), thapsigargin (TG, an inhibitor of ER Ca^2+^-ATPase), and an antibody against β-actin were purchased from Sigma-Aldrich (St. Louis, MO, USA). Antibodies against TonEBP and lamin-B1 were obtained from Abcam (Cambridge, UK), and antibodies against p-nuclear factor of kappa light polypeptide gene enhancer in B cells inhibitor alpha (Iκ-Bα)/Iκ-Bα, p-p38/p38, p-ERK/ERK, p-JNK/JNK, and p-protein kinase C (PKC)/PKC were purchased from Cell Signaling Technology (Danvers, MA, USA). Horseradish peroxidase (HRP)-conjugated secondary antibodies were obtained from Santa Cruz Biotechnology (Santa Cruz, CA, USA). Small interfering RNAs (siRNAs) against TonEBP and TLR-2 were purchased from Bioneer (Daejeon, South Korea). Antibodies against MMPs 1, 3, and 13 were obtained from R&D Systems (Minneapolis, MN, USA).

### Cartilage collection and chondrocyte isolation from cartilage

OA cartilage samples were obtained from the knee joints of OA patients (n = 9, 71.2 ± 3.6 years of age) at the time of total knee replacement surgery. Patient diagnoses were determined using the criteria developed by the American College of Rheumatology. Normal cartilage samples were obtained from the femoral heads of patients (n = 6, 70.7 ± 12.5 years of age) with femoral neck fractures and no known history of OA or rheumatoid arthritis (RA). The collection and use of human tissue samples were reviewed and approved by the Institutional Review Board of Hallym University Sacred Heart Hospital, Anyang, South Korea (approval number 2018-05-040). All patients provided written informed consent for the use of their discarded cartilage samples. All methods were carried out in accordance with relevant guidelines and regulations.

### Quantitative reverse-transcription polymerase chain reaction (qRT-PCR)

Total RNA was isolated from chondrocytes using TRIzol reagent. cDNA was synthesized from total RNA using Moloney murine leukemia virus reverse transcriptase (Promega, Madison, WI, USA). The qRT-PCR reaction mix contained SYBR Green PCR master mix, forward and reverse primers, and an equal amount of cDNA. qRT-PCR was performed using the StepOnePlus Real-Time PCR system (Applied Biosystems, Foster city, CA, USA). The primer sequences used for qRT-PCR (Table S1) were obtained from Cosmo Genetech Co. (Seoul, South Korea).

### Fluorescence microscopy analysis

To detect the level of TonEBP in chondrocytes, cells were seeded on Nunc Lab-Tek II chamber slides and treated with 29-kDa FN-f for 24 h. The cells were fixed in 4% paraformaldehyde at room temperature for 10 min then sequentially incubated with primary rabbit anti-TonEBP (1: 1000 or 500 dilution) overnight at 4 °C, followed by Alexa Fluor 588-conjugated goat anti-rabbit secondary antibody for 30 min at room temperature. Nuclei were counterstained with 4′,6′-diamidino-2-phenylindole (DAPI, 1 µg/mL; Roche Applied Science, Basel, Switzerland), followed by visualization under fluorescence microscopy (Eclipse Ni-E; Nikon, Tokyo, Japan).

### Western blot analysis

Cells were lysed in RIPA buffer (Biosesang, Gyeonggi, South Korea), and equal amounts of protein samples were separated by 10% or 15% sodium dodecyl sulfate–polyacrylamide gel electrophoresis and electroblotted onto a polyvinylidene difluoride membrane (Bio-Rad Laboratories, Hercules, CA, USA). The membranes were blocked with 5% nonfat milk in Tris-buffered saline plus 0.01% Tween 20 and sequentially incubated with primary and secondary antibodies. The antibody complexes on the membrane were visualized using an enhanced chemiluminescence kit.

### siRNA knockdown of TonEBP and TLR-2

To form siRNA–liposome complexes, the siRNA, which was gently mixed with plus reagent and serum-free medium, was added to a solution containing Lipofectamine 2000 reagent and serum-free medium and incubated for 15 min at room temperature. The mixture containing siRNA–liposome complexes was added to chondrocytes, and the cells were incubated for 3 h at 37 °C and then maintained with fresh medium for 48 h prior to treatment with 29-kDa FN-f. The cells were incubated with 29-kDa FN-f (300 nM) for 6 or 24 h for qRT-PCR and western blot analyses, respectively. The sequence of siRNA are shown in Table S2.

### Enzyme-linked immunosorbent assay (ELISA) of MMP-13

Culture medium (100 µL) from 29-kDa FN-f-treated and untreated chondrocytes was added to wells of round-bottom microtiter plates and incubated for 14 h at 4 °C. Anti-MMP-13 antibody (100 µL/well, 1:1000 dilution) was added to each well for 14 h at 4 °C, followed by incubation with HRP-conjugated secondary antibody for 1 h. Then, antibody complexes were exposed to 3,3′,5,5′-tetramethylbenzidine liquid substrate ELISA reagent (Sigma Aldrich) for 30 min, and then stop solution was added to each well. The absorbance was measured at 450 nm.

### Nuclear translocation of TonEBP

To examine translocation of TonEBP into the nucleus, ice-cold cytoplasmic extraction buffer was added to cell pellets. After centrifugation at 14,000×*g*, the supernatant (cytoplasmic fraction) was transferred to a clean pre-chilled tube. The insoluble fraction was treated with ice-cold nuclear extraction reagent and vortexed for 15 s every 10 min for 40 min. The supernatant (nuclear fraction) was obtained by centrifugation. Cytoplasmic and nuclear fractions were subjected to western blot analysis.

### Measurement of intracellular calcium ion (Ca^2+^) influx

Intracellular Ca^2+^ concentrations were measured using the Fluo-8 No Wash Calcium Assay kit (AbCam) according to the manufacturer’s instructions. Briefly, primary chondrocytes were seeded into a black-walled clear-bottom 96-well plate at 1 × 10^5^ cells/well and cultured in serum-free medium for 24 h. Cells were preloaded with Fluo-8 dye-loading solution (50 µL/well) for 30 min at 37 °C and incubated for another 30 min at room temperature. Then, 29-kDa FN-f or phosphate-buffered saline (PBS) was injected into each well via a dispenser in the Synergy Mx microplate reader (BioTek instruments, Winooski, VT, USA). Fluorescence intensity was monitored for 90 s at an excitation/emission of 490/525 nm. A23187, a Ca^2+^ ionophore, was additionally injected into each well, and the fluorescence was measured for 90 s at 490/525 nm.

### Statistical analysis

Differences between two groups were evaluated using the nonparametric Mann–Whitney *U* test. Statistical analyses were performed using GraphPad Prism 6.07 (GraphPad Software, San Diego, CA, USA, https://www.graphpad.com/scientific-software/prism/). Data are expressed as means ± standard deviation (SD). A *P*-value < 0.05 was considered to indicate statistical significance.

## Results

### TonEBP levels were increased in OA cartilage and 29-kDa FN-f-treated chondrocytes

**Figure 1:**
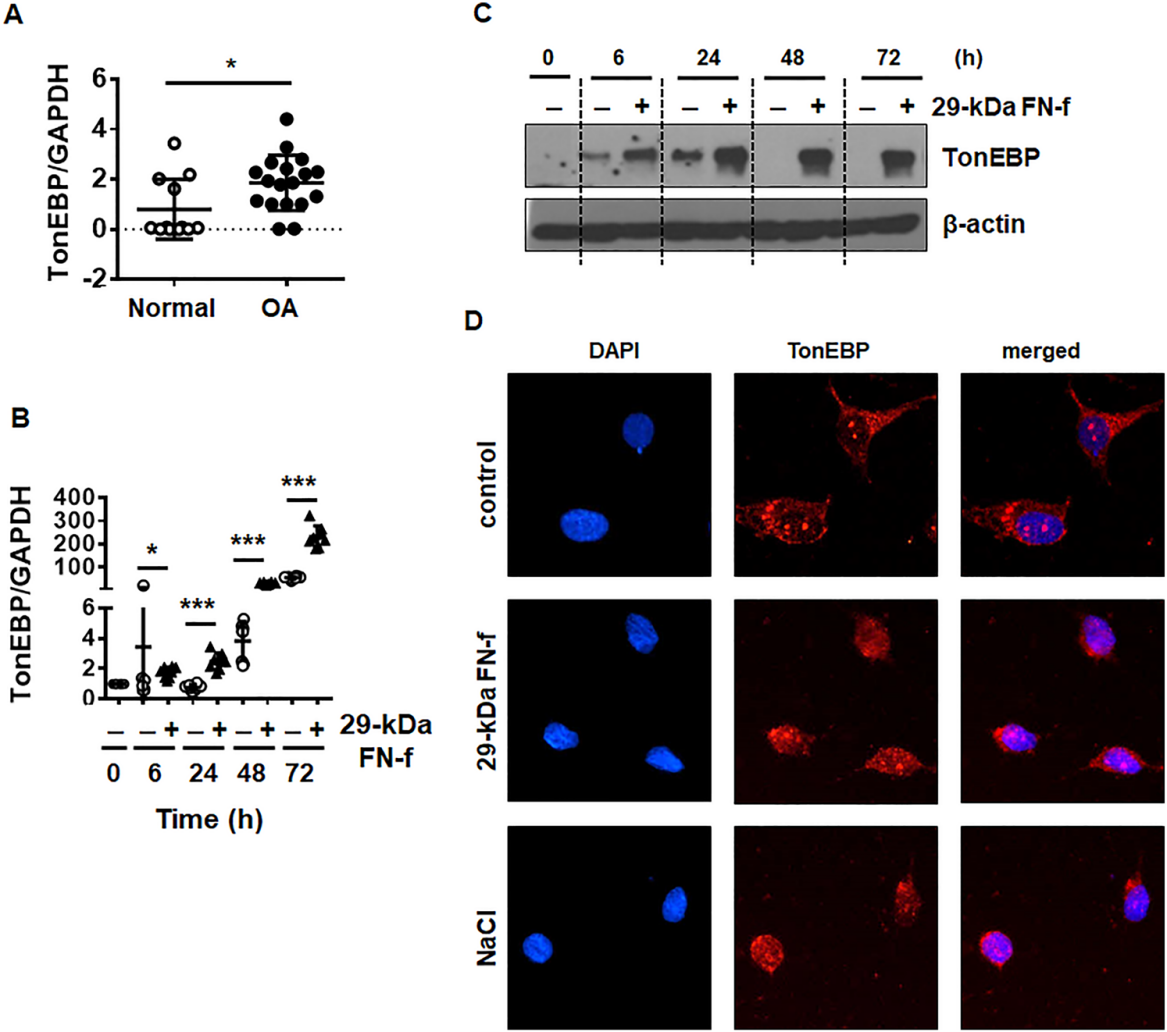
29-kDa FN-f induces TonEBP expression in chondrocytes. (**A**) The level of TonEBP was elevated in OA compared with normal cartilage. The relative expression of TonEBP in normal and OA cartilage was determined via QuantiFast SYBR Green qRT-PCR. The expression level of TonEBP relative to that of glyceraldehyde 3-phosphate dehydrogenase (GAPDH), the internal control, is shown. Data represent the mean ± SD of duplicate data from six normal and nine OA donors. *P < 0.05 vs. normal cartilage. (**B**) mRNA and (**C**) protein levels of TonEBP were significantly increased in a time-dependent manner after treatment with 29-kDa FN-f. Chondrocytes were stimulated with 29-kDa FN-f (300 nM) for 6, 24, 48, and 72 h. PCR data are expressed as the mean ± SD of duplicate data from four independent donors. *P < 0.05 and ***P < 0.001 vs. untreated control. (**C**) The western blot shown is representative of blots from three independent donors. (**D**) Nuclear localization of TonEBP was induced by 29-kDa FN-f. After stimulation of primary chondrocytes with 29-kDa FN-f or NaCl (110 mM) for 24 h, nuclear levels of TonEBP were detected by fluorescence microscopy. Nuclei were stained with DAPI. (**A**,**B**) The figures were analyzed and produced by GraphPad Prism software (GraphPad Prism 6.07; https://www.graphpad.com/scientific-software/prism/).

To investigate whether the level of TonEBP is related to the pathogenesis of OA, TonEBP expression was compared between normal and OA cartilage. The mRNA levels of TonEBP in OA cartilage, which exhibited high MMP-13 level and low Col II level (Fig. S1), were significantly increased compared with normal cartilage (Fig. 1A).

Next, we examined whether 29-kDa FN-f influences TonEBP expression at the mRNA and protein levels. TonEBP mRNA was significantly upregulated from 6 h after treatment with 29-kDa FN-f in a time-dependent manner (Fig. 1B). Western blot analysis showed that the TonEBP protein significantly increased after 6 h of treatment with 29-kDa FN-f (Fig. 1C). Notably, the TonEBP mRNA level also increased in control chondrocytes in a time-dependent manner; however, this increase was not accompanied by an increase in protein expression. Immunofluorescence showed that TonEBP was predominantly localized in the cytoplasm in untreated chondrocytes (Fig. 1D). Treatment with 29-kDa FN-f increased the nuclear localization of TonEBP to a degree comparable with that observed in a positive control induced by hyperosmolarity (NaCl, 110 mM) (Fig. 1D). These results demonstrate that expression of TonEBP increased in OA and 29-kDa FN-f-treated chondrocytes.

### TonEBP influences 29-kDa FN-f-dependent expression of MMPs

**Figure 2:**
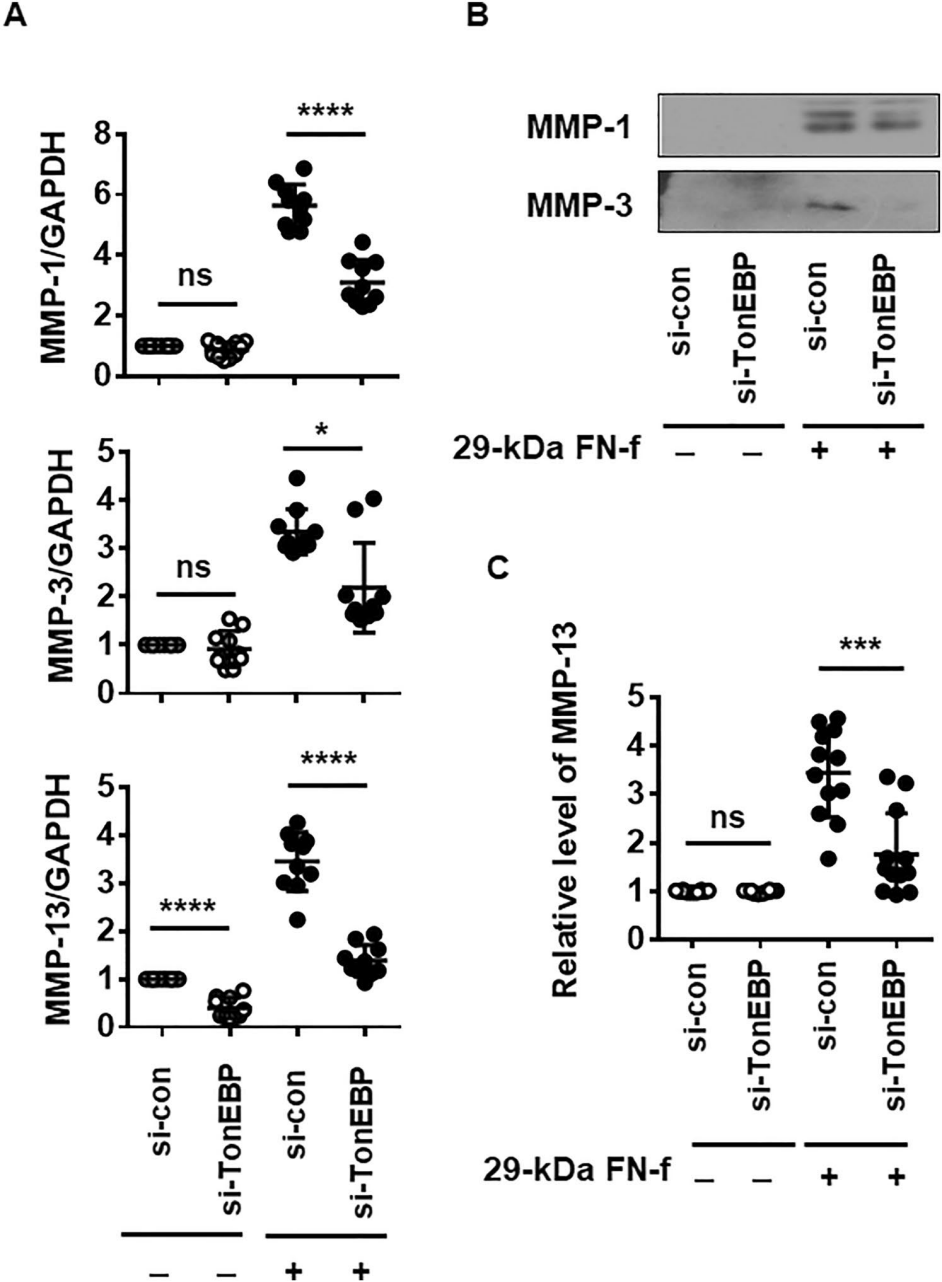
TonEBP regulates 29-kDa FN-f-dependent expression of MMPs. Knockdown of TonEBP suppressed MMP expression induced by 29-kDa FN-f at both the (**A**) mRNA and (**B**,**C**) protein levels. Chondrocytes were transfected with si-TonEBP. After 48 h, cells were stimulated with 29-kDa FN-f (300 nM) for (**A**) 6 h and (**B**,**C**) 24 h. mRNA levels of MMPs 1, 3, and 13 were measured by QuantiFast SYBR Green qRT-PCR. Data are expressed as the mean ± SD of duplicate data from five independent donors. *P < 0.05 and ****P < 0.0001 vs. si-con-treated control; ns, not significant. (**B**) Protein expression of MMPs 1 and 3 from chondrocyte culture medium were measured by western blot analysis. (**C**) MMP-13 protein expression was measured by ELISA. Culture medium from 29-kDa FN-f-treated and untreated cells was incubated in a round-bottom microplate for 14 h, and then an antibody against MMP-13 was added to each well followed by further incubation for 14 h. Data are expressed as the mean ± SD of triplicate data from four independent donors. ***P < 0.001 vs. si-con-treated control; ns, not significant. (**A**,**C**) The figures were analyzed and produced by GraphPad Prism software (GraphPad Prism 6.07; https://www.graphpad.com/scientific-software/prism/).

29-kDa FN-f induces MMP expression via the TLR-2/myeloid differentiation primary response 88 signaling pathway^13^. To investigate whether TonEBP is involved in 29-kDa FN-f-induced MMP expression, knockdown of TonEBP was performed by transfection with an siRNA targeting TonEBP (si-TonEBP). 29-kDa FN-f highly increased expression of MMPs 1, 3, and 13 at both the mRNA and protein levels, whereas TonEBP silencing significantly reduced 29-kDa FN-f-stimulated MMP expression (Fig. 2A–C). In addition, 29-kDa FN-f suppressed tissue inhibitor of matrix metalloproteinase-1 (TIMP-1) expression in a time-dependent manner and TonEBP silencing recovered 29-kDa FN-f-suppressed TIMP-1 expression (Fig. S2A and B). These results demonstrate that TonEBP is a regulator of 29-kDa FN-f-induced MMP expression.

### Activation of mitogen-activated protein kinase (MAPK)/NF-κB signaling via PLC-γ contributes to 29-kDa FN-f-dependent nuclear translocation of TonEBP

**Figure 3:**
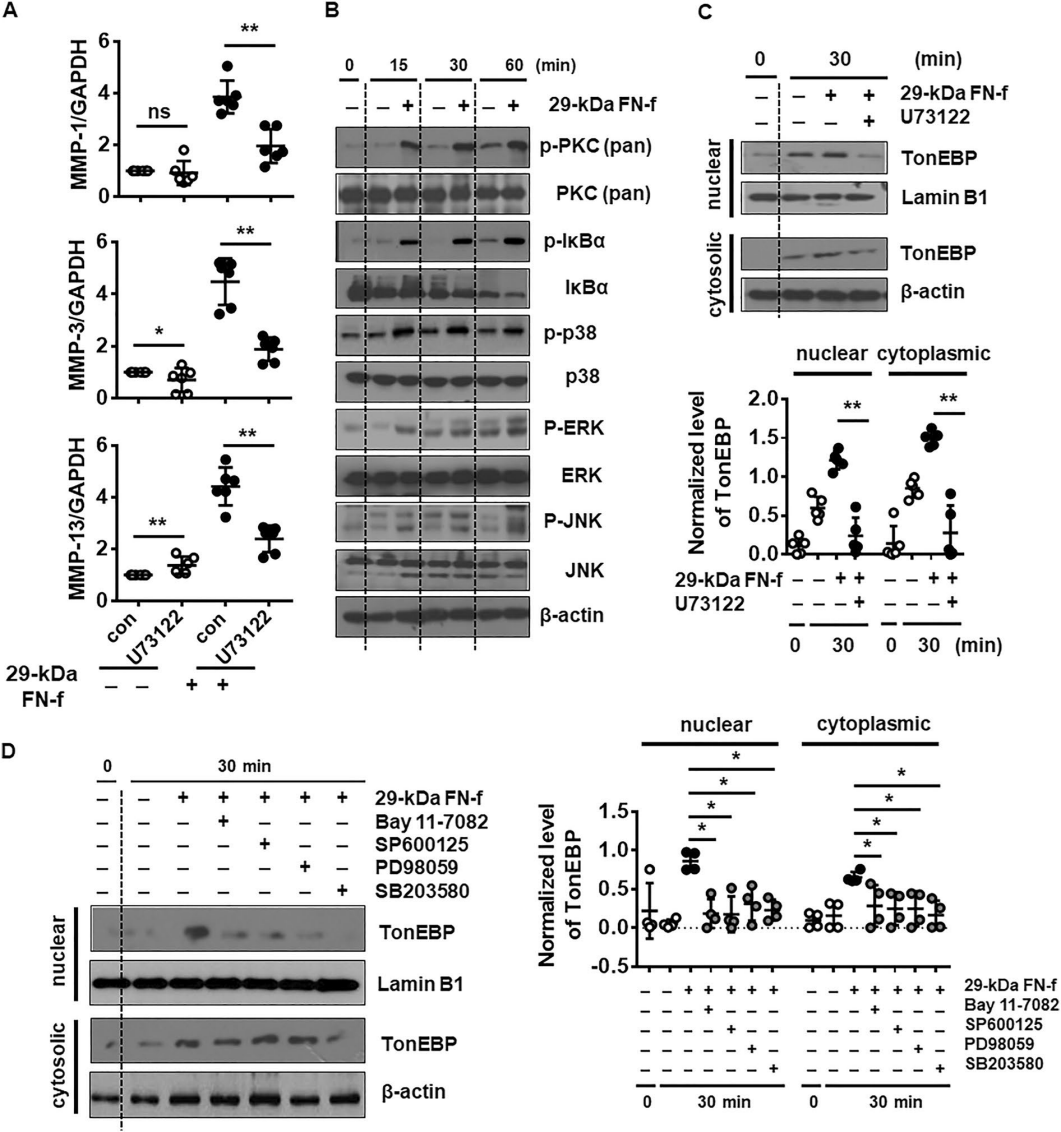
29-kDa FN-f induces nuclear accumulation of TonEBP via the PLC/NF-κB/MAPK signaling pathway. (**A**) 29-kDa FN-f-induced MMP expression was suppressed by U73122 (a PLC-γ-specific inhibitor). Chondrocytes were pretreated with U73122 for 2 h and incubated with 29-kDa FN-f (300 nM) for 6 h. Expression levels of MMPs 1, 3, and 13 were measured by QuantiFast SYBR Green qRT-PCR. Data are expressed as the mean ± SD of duplicate data from three independent donors. *P < 0.05 and **P < 0.01 vs. untreated control; ns, not significant. (**B**) 29-kDa FN-f activated PKC, NF-κB, and MAPK signaling. Chondrocytes were treated with 29-kDa FN-f (300 nM) for 15, 30, and 60 min. The levels of p-PKC/PKC, p-Iκ-Bα/Iκ-Bα, p-p38/p38, pERK/ERK, and p-JNK/JNK were determined by western blot analysis. (**C**) 29-kDa FN-f increased nuclear accumulation of TonEBP via the PLC-γ signaling pathway. Cytoplasmic and nuclear fractions were isolated from chondrocytes treated with 29-kDa FN-f (300 nM) for 30 min after pre-treatment with U73122 for 2 h. The western blot shown is representative of blots from five independent donors. (**D**) Inhibitors of NF-κB and MAPK signaling decreased 29-kDa FN-f-induced nuclear translocation of TonEBP. Chondrocytes were pre-incubated with a variety of inhibitors for 2 h and stimulated with 29-kDa FN-f (300 nM) for 30 min. The level of TonEBP in nuclear and cytoplasmic fractions from cell lysates was measured by western blot analysis. The western blot shown is representative of blots from four independent donors. (**C**,**D**) Protein band density in western blot image was assessed by setting a threshold using Image J software version 1.51 (National Institutes of Health, Bethesda, MD, USA, https://imagej.nih.gov/ij/download.html). Levels of nuclear and cytoplasmic TonEBP were normalized to lamin B1 and β-actin, respectively. Data are expressed as the mean ± SD of band densities of western blots from four or five different donors. *P < 0.05 and **P < 0.01 vs. 29-kDa FN-f-treated cells. U73122 (1 µM), PLC-γ inhibitor; Bay 11-7082 (5 µM), NF-κB inhibitor; SP6001250 (10 µM), JNK inhibitor; PD98059 (10 µM), MEK1/2 inhibitor; SB203580 (1 µM), p38 inhibitor. (**A**,**C**,**D**) The figures were analyzed and produced by GraphPad Prism software (GraphPad Prism 6.07; https://www.graphpad.com/scientific-software/prism/).

The PLC-γ/PKC axis activates several signaling pathways to regulate the expression of ECM-related genes, including MMP-13 and type II collagen (Col II), in human OA chondrocytes^16^. PLC-γ hydrolyzes phosphatidylinositol biphosphate to diacylglycerol and inositol 1,4,5-trisphosphate, which in turn allow activation of PKC and intracellular mobilization of Ca^2+^, respectively^17^. To elucidate whether PLC-γ activation is involved in 29-kDa FN-f-dependent expression of TonEBP, chondrocytes were treated with 29-kDa FN-f in combination with U73122 (1 µM). First, inhibition of PLC-γ significantly suppressed 29-kDa FN-f-induced expression of MMPs 1, 3, and 13 (Fig. 3A). In addition, activation of downstream signaling molecules of PLC-γ, including PKC, p38, ERK, JNK, and Iκ-Bα, was rapidly increased at 15 min and highly maintained until 60 min after treatment with 29-kDa FN-f (Fig. 3B). Our data showed that the PLC-γ signaling axis was related to expression of MMPs and accompanied by activation of MAPK/NF-κB signaling induced by 29-kDa FN-f.

Next, activation (nuclear translocation) of TonEBP protein was examined 30 min after treatment with 29-kDa FN-f in the presence of U73122 (1 µM) for 2 h. Both 29-kDa FN-f-dependent nuclear translocation and cytoplasmic expression of TonEBP were reduced by addition of U73122 (Fig. 3C). In addition, JNK/ERK/p38 MAPK and Iκ-Bα inhibitors significantly suppressed TonEBP nuclear translocation as well as cytoplasmic expression (Fig. 3D). Therefore, these results demonstrate that 29-kDa FN-f promotes nuclear localization of TonEBP via the PLC-γ/PKC/MAPK and NF-κB signaling pathways.

### TLR-2 is responsible for 29-kDa FN-f-dependent activation of TonEBP

**Figure 4:**
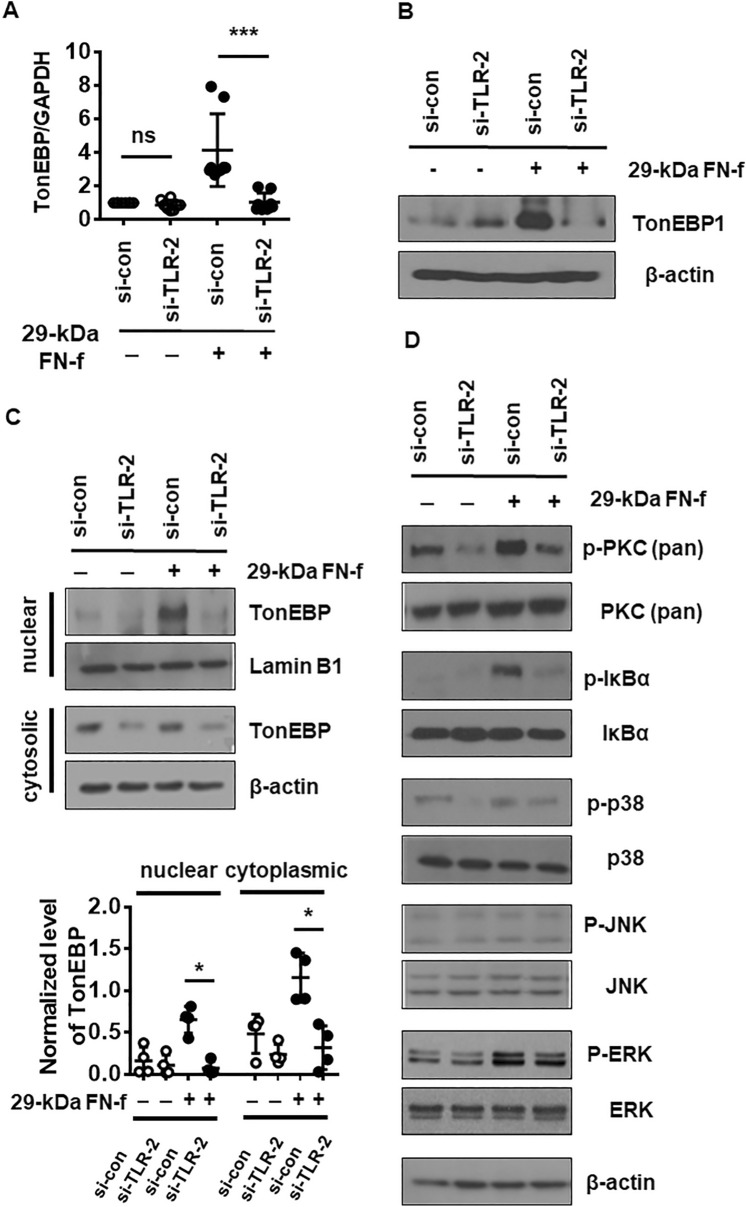
TLR-2 is responsible for 29-kDa FN-f-induced TonEBP expression and nuclear translocation. (**A**,**B**) Knockdown of TLR-2 decreased 29-kDa FN-f-induced expression of TonEBP at both the (**A**) mRNA and (**B**) protein levels. Chondrocytes were transfected with si-TLR-2 and si-control followed by treatment with 29-kDa FN-f (300 nM) for 6 h (mRNA) or 24 h (protein). mRNA and protein levels of TonEBP were measured by qRT-PCR and western blot analysis, respectively. β-actin served as a loading control. PCR data show the mean ± SD of duplicate experiments in chondrocytes from four different donors. ***P < 0.001 vs. si-con-transfected control; *ns* not significant. (**C**) TLR-2 knockdown affected nuclear translocation of TonEBP. Protein band density in western blot image was assessed by setting a threshold using Image J software version 1.51 (National Institutes of Health, Bethesda, MD, USA, https://imagej.nih.gov/ij/download.html). Levels of nuclear and cytoplasmic TonEBP were normalized to lamin B1 and β-actin, respectively. Data are expressed as the mean ± SD of band densities on western blots from four different donors (n = 4). *P < 0.05 vs. si-con-treated cells. (D) TLR-2 knockdown suppressed activation of PKC, p-38 MAPK, and NF-κB. Chondrocytes were transfected with si-TLR-2. After 48 h, cells were stimulated with 29-kDa FN-f (300 nM) for 30 min. The western blot shown is representative of blots from four independent donors. (**A**,**C**) The plots were analyzed and produced by GraphPad Prism software (GraphPad Prism 6.07; https://www.graphpad.com/scientific-software/prism/).

We previously demonstrated that 29-kDa FN-f signals via TLR-2 to mediate catabolic and anabolic responses^13,18^. To examine the role of TLR-2 in 29-kDa FN-f-mediated TonEBP expression, chondrocytes were transfected with an siRNA targeting TLR-2 (si-TLR-2), followed by treatment with 29-kDa FN-f. TLR-2 knockdown significantly decreased 29-kDa FN-f-induced TonEBP expression at both the mRNA and protein levels (Fig. 4A,B). Furthermore, silencing of TLR-2 significantly decreased nuclear accumulation of TonEBP as well as its cytoplasmic expression (Fig. 4C). In turn, knockdown of TLR-2 apparently abolished 29-kDa FN-f-stimulated activation of PKC, p38 MAPK, and Ik-Bα (Fig. 4D). These results demonstrate that 29-kDa FN-f signals via TLR-2 to induce TonEBP expression and its nuclear translocation.

### 29-kDa FN-f modulated expression of osmoregulatory genes and voltage-dependent calcium channels (VDCCs) via TLR-2/TonEBP signaling

**Figure 5:**
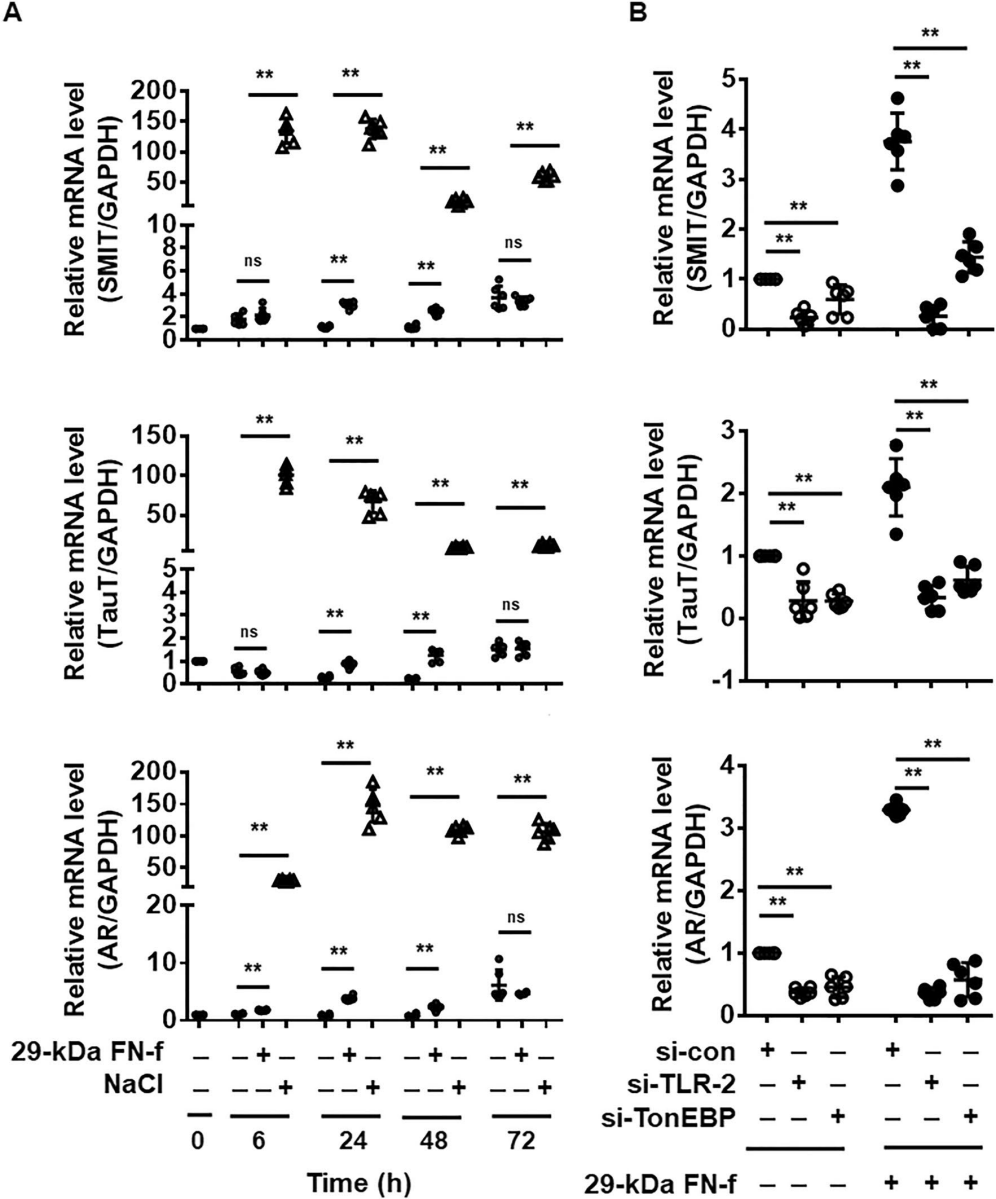
29-kDa FN-f induces osmoregulatory gene expression via TLR-2/TonEBP signaling. (**A**) 29-kDa FN-f affected the expression of SMIT, TauT, and AR. Chondrocytes were stimulated with 29-kDa FN-f (300 nM) or NaCl (110 mM) for 6, 24, 48, and 72 h. (**B**) TLR-2/TonEBP signaling was involved in 29-kDa FN-f-induced expression of osmoregulatory genes. Knockdown of TLR-2 or TonEBP was performed by incubating chondrocytes with TLR-2 or TonEBP siRNA for 48 h. After treatment with 29-kDa FN-f for 24 h, SMIT, TauT, and AR mRNA levels were measured by QuantiFast SYBR Green qRT-PCR. Data are expressed as the mean ± SD of duplicate data from three independent donors. **P < 0.01 vs. untreated or si-con-treated control; ns, not significant. All figures were analyzed and produced by GraphPad Prism software (GraphPad Prism 6.07; https://www.graphpad.com/scientific-software/prism/).

TonEBP regulation and downstream signaling differ according to the stimulus. Hyperosmolarity (NaCl) increases cellular expression and nuclear translocation of TonEBP, which induces the expression of osmoregulatory genes and heat-shock proteins, leading to prevention of cellular damage, whereas TNF-α induces nuclear accumulation of TonEBP but not expression of osmoregulatory genes^12^. We investigated whether 29-kDa FN-f induces expression of osmoregulatory genes, including taurine transporter (*TauT/SLC6A6*), sodium/myo-inositol cotransporter (*SMIT/SLC5A3*), and aldose reductase (*AR/AKR1B1*). As expected, osmoregulatory genes were highly upregulated from 6 h after stimulation with NaCl, persisting for 72 h (Fig. 5A). Interestingly, osmoregulatory genes were slightly but significantly induced by treatment with 29-kDa FN-f for 24 and 48 h, although at a much lower level than that induced by NaCl treatment (Fig. 5A). These data show that 29-kDa FN-f affected the expression of osmoregulatory genes, although its induction was weak and delayed. We examined whether 29-kDa FN-f induces expression of TauT, SMIT, and AR via TLR-2/TonEBP. TLR-2- or TonEBP-silenced chondrocytes were exposed to 29-kDa FN-f for 24 h. PCR analysis showed that increased expression of TauT, SMIT, and AR by 29-kDa FN-f were significantly suppressed in TLR-2- and TonEBP-knockdown cells (Fig. 5B). TLR-2- and TonEBP knockdown also decreased TauT, SMIT, and AR in untreated chondrocytes. These results demonstrate that 29-kDa FN-f can induce osmoregulatory gene expression via the TLR-2/TonEBP signaling pathway.

**Figure 6:**
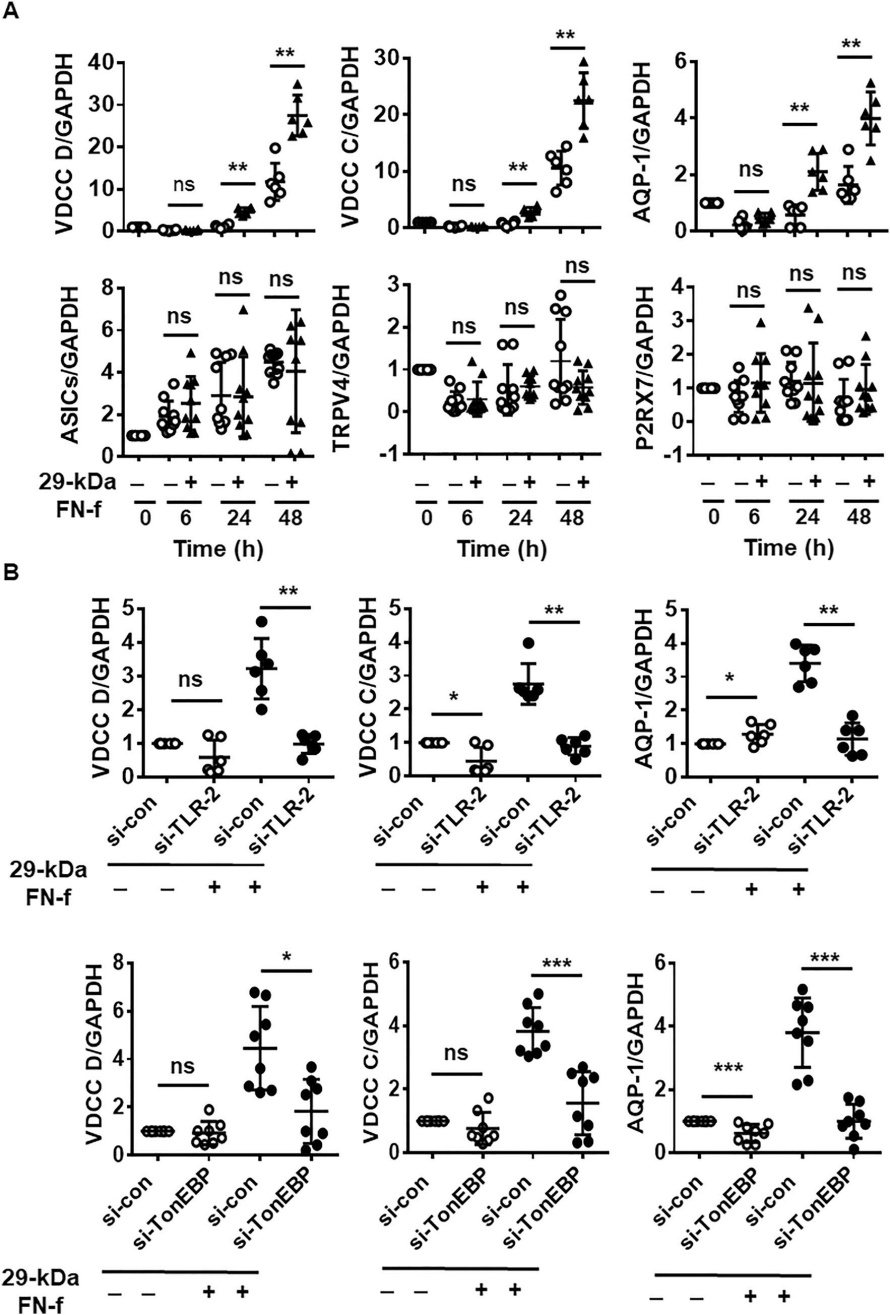
29-kDa FN-f increases TonEBP-dependent expression of VDCCs and AQP-1. (**A**) Effect of 29-kDa FN-f on expression of VDCCs (CACNA1C and CACNA1D), AQP-1, ASIC, TRPV4, and P2RX7. Chondrocytes were stimulated with 29-kDa FN-f (300 nM) for 6, 24, and 48 h. (**B**) Effect of TLR-2/TonEBP signaling on induction of VDCCs and AQP-1. Chondrocytes were incubated with si-TLR-2 or si-TonEBP for 48 h followed by treatment with 29-kDa FN-f for 24 h. Data are expressed as the mean ± SD of duplicate data from more than three independent donors. *P-*values represent comparisons between two groups. *P < 0.05, **P < 0.01, and ***P < 0.001 vs. untreated or si-con-treated control; ns, not significant. All figures were analyzed and produced by GraphPad Prism software (GraphPad Prism 6.07; https://www.graphpad.com/scientific-software/prism/).

In response to osmolarity, expression of aquaporin 2, a tonicity-sensitive water channel, is regulated in a TonEBP-dependent, but CaN pathway-independent manner, in NP chondrocytes, indicating that they can readily respond to an applied stimulus such as hyperosmolarity via regulation of channel expression^19^. We examined whether 29-kDa FN-f could alter the expression of ion channels, including VDCCs (Ca_v_1.2 and Ca_v_1.3), aquaporin-1 (AQP-1), acid-sensing ion channel (ASIC), transient receptor potential cation channel subfamily V 4 (TRPV4), and purinergic receptor P2X 7 (P2RX7). The expression of VDCCs and AQP-1 was significantly increased by treatment with 29-kDa FN-f at 24 h and remained high until 48 h. Their expression levels were also increased in untreated chondrocytes after 72 h of culture (Fig. 6A). By contrast, the expression levels of ASIC, TRPV4, and P2RX7 were not affected by 29-kDa FN-f (Fig. 6A). Thus, these data demonstrate that prolonged treatment with 29-kDa FN-f modulates expression of VDCCs and AQP-1. To examine whether TLR-2/TonEBP signaling influences the expression of VDCCs and AQP-1, TLR-2 in chondrocytes was knocked down by transfection with si-TLR-2 and si-TonEBP. 29-kDa FN-f-induced expression of VDCCs, and AQP-1 was significantly suppressed by silencing of TLR-2 and TonEBP (Fig. 6B), indicating that expression of VDCCs and AQP-1 was significantly increased by 29-kDa FN-f via the TLR-2/TonEBP signaling pathway. These results demonstrate that 29-kDa FN-f regulates expression of osmoregulatory genes and channels related to osmolarity via TLR-2/TonEBP.

### 29-kDa FN-f-induced MMP expression is dependent on Ca^2+^/calmodulin (CaM)/calcineurin (CaN) signaling

**Figure 7:**
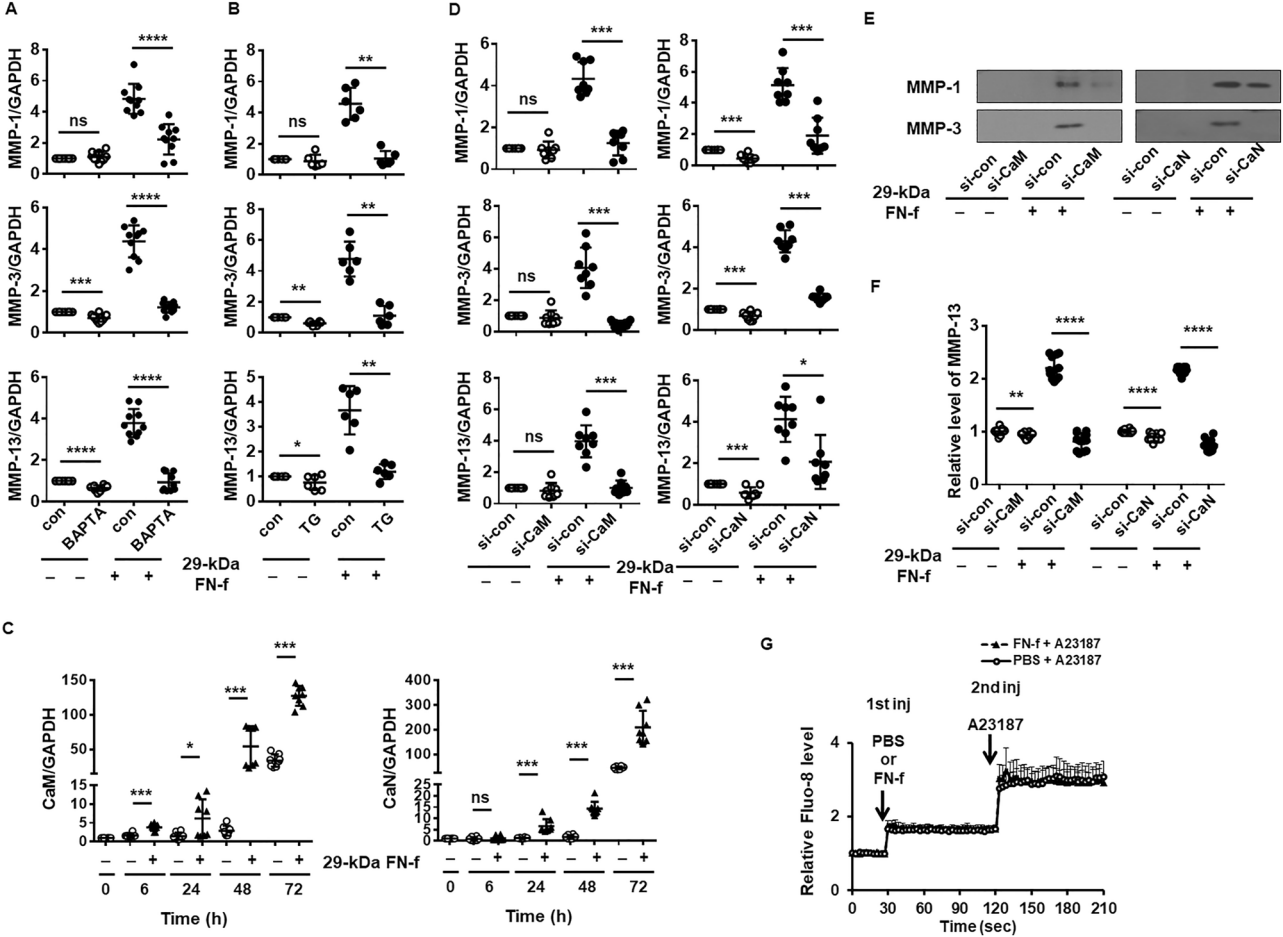
29-kDa FN-f induces MMP expression via the CaN-dependent signaling pathway. (**A**,**B**) Depletion of the intracellular calcium concentration reduced MMP expression by 29-kDa FN-f. Chondrocytes were pretreated with (**A**) BAPTA, a calcium chelator, and (**B**) TG, an inhibitor of the sarcoendoplasmic reticulum Ca^2+^ ATPase (SERCA) for 2 h and then incubated with 29-kDa FN-f for 6 h. Data are expressed as the mean ± SD of duplicate data from more than three independent donors (n ≥ 3). *P-*values represent comparisons between two groups. *P < 0.05, **P < 0.01, ***P < 0.001, and ****P < 0.0001 vs. BAPTA- or TG-untreated cells; ns, not significant. (**C**) 29-kDa FN-f affected CaM and CaN expression. Cells were treated with 29-kDa FN-f for 6, 24, 48, and 72 h. The levels of CaM and CaN were measured by QuantiFast SYBR Green qRT-PCR. Data are expressed as the mean ± SD of duplicate data from four independent donors. *P < 0.05 and ***P < 0.001 vs. untreated control; ns, not significant. (**D**) Knockdown of CaM and CaN reduced MMP expression induced by 29-kDa FN-f. Chondrocytes were incubated with si-CaM and si-CaN for 48 h, followed by treatment with 29-kDa FN-f for 6 h. Expression levels of MMPs 1, 3, and 13 were measured by QuantiFast SYBR Green qRT-PCR. Data are expressed as the mean ± SD of duplicate data from four independent donors (n = 4). *P-*values represent comparisons between two groups. (**E**,**F**) The protein levels of MMPs 1, 3, and 13 in culture medium from 29-kDa FN-f-stimulated chondrocytes were measured by (**E**) western blot and (**F**) ELISA. The western blot shown is representative of blots from three independent donors (n = 3). *P < 0.05, **P < 0.01, ***P < 0.001, and ****P < 0.0001 vs. si-con-treated control; ns, not significant. (**G**) 29-kDa FN-f induced no change in the intracellular Ca^2+^ concentration. Chondrocytes were pretreated with Fluo-8 dye loading solution for 30 min at 37 °C and then incubated for 30 min at room temperature, followed by the first injection of 29-kDa FN-f (300 nM). The fluorescence intensity was monitored at 490/525 nm for 90 s using a Synergy Mx microplate reader. Then, A23187 ionophore was injected into the cells, followed by monitoring the fluorescence intensity for 90 s. Data are expressed as the mean ± SD of triplicate data from two independent experiments. (**A**–**D**) and (**F**,**G**) The figures were analyzed and produced by GraphPad Prism software (GraphPad Prism 6.07; https://www.graphpad.com/scientific-software/prism/).

In response to stimuli, activation of PLC and generation of inositol 1,4,5-trisphosphate allow rapid transient release of Ca^2+^ from endoplasmic reticulum (ER) intracellular stores, which activates CaN, a Ca^2+^/CaM-activated phosphatase, subsequently leading to dephosphorylation and nuclear localization of NFAT proteins. In addition, diacylglycerol, another second messenger produced by PLC, activates PKC in several signaling pathways^20,21^. To confirm whether 29-kDa FN-f-induced MMP expression was altered following intracellular calcium perturbation, 29-kDa FN-f-induced MMP expression was measured in the presence of BAPTA or TG. BAPTA-induced depletion of intracellular Ca^2+^, and TG-induced depletion of ER Ca^2+^ storage significantly suppressed 29-kDa FN-f-induced expression of MMPs 1, 3, and 13 (Fig. 7A,B), showing that persistent perturbation of Ca^2+^ inhibits 29-kDa FN-f-induced MMP expression. These results suggest that 29-kDa FN-f can regulate MMP expression via a Ca^2+^-dependent mechanism. To investigate whether 29-kDa FN-f-induced MMP expression is dependent of CaM/CaN signaling, we first measured the expression of CaM and CaN following exposure to 29-kDa FN-f. Expression of both CaM and CaN was precipitously increased from 48 h after treatment with 29-kDa FN-f (Fig. 7C). CaM and CaN were also increased in the control culture after 72 h. 29-kDa FN-f significantly increased expression of MMPs 1, 3, and 13 at both the mRNA and protein levels (Fig. 7D–F). Silencing of CaM and CaN significantly suppressed 29-kDa FN-f-stimulated expression of MMPs 1, 3, and 13 (Fig. 7D). These results suggest that in addition to the TonEBP signaling pathway, the CaM/CaN signaling pathway can at least partially affect 29-kDa FN-f-induced MMP expression as well.

We next measured the change in the intracellular Ca^2+^ concentration induced by 29-kDa FN-f using the Fluo-8 Calcium Assay kit. Intracellular influx of Ca^2+^ was similar between chondrocytes treated with PBS and those treated with 29-kDa FN-f (Fig. 7G). The Ca^2+^ ionophore A23187, which was used as a positive control, significantly increased intracellular Ca^2+^ concentrations in both 29-kDa FN-f- and PBS-treated cells (Fig. 7G), indicating that 29-kDa FN-f did not directly induce Ca^2+^ influx.

## Discussion

TonEBP/NFAT5 is a transcription factor responsible for hyperosmolarity-induced expression of osmoregulatory genes as well as TNF-α-induced inflammatory responses^5,12^. In this study, we demonstrated the function and activity of the TonEBP signaling pathway in 29-kDa FN-f-induced catabolic factor expression. Human OA cartilage showed high expression of TonEBP compared with normal cartilage. 29-kDa FN-f induced MMP expression together with increased expression and nuclear translocation of TonEBP. Notably, prolonged incubation with 29-kDa FN-f resulted in increased expression of osmoregulatory genes and VDCCs. Furthermore, the CaM/CaN signaling pathway was also involved in 29-kDa FN-f-induced MMP expression. These results demonstrate that 29-kDa FN-f induces cartilage ECM catabolism via transcription factor TonEBP in addition to weak but significant osmoregulatory signaling.

Proteoglycans in cartilage tissues are composed of core proteins and abundantly attached glycosaminoglycans, which are highly negatively charged and bind to a variety of cations, including sodium, potassium, and calcium, as well as water. Cartilage is hyperosmolar compared with other tissues, and gradual loss of cartilage matrix in degenerative joint diseases leads to an increase in osmolarity. Fibronectin (FN) is an important component of articular cartilage matrix, with its degradation product, FN-fs, present abundantly in OA synovial fluids^22,23^. FN-fs are potent catabolic mediators with as strong effect as inflammatory cytokines, thus, working as an innate molecule of damage-associated molecular pattern^13,15,18,24^. In addition, loss of extracellular matrix by FN-f-induced cartilage damage reduces binding of cationic ions to negatively charged proteoglycans and increases synovial osmolarity, which increases expression of TonEBP and its target molecules, including MMPs, subsequently forming vicious cycle of aggravating the pathogenesis of OA.

Previous studies revealed that hyperosmolarity affects matrix synthesis in articular cartilage and the intervertebral disc, such that increases in osmolarity by addition of NaCl, sucrose, or polyethylene glycol in vitro stimulated proteoglycan synthesis in intervertebral disc cartilage^25–27^. Similarly, in bovine intervertebral disc chondrocytes, hyperosmolarity elevated matrix protein expression, together with inhibition of MMP-3 expression^28^. On the other hand, hyperosmotic pressure downregulated ACAN gene expression as well as cell size in articular cartilage chondrocytes, while the physiological ionic strength of native cartilage (350–400 mOsm) induced a maximal rate of matrix synthesis^29–31^. In response to hyperosmolarity, mRNA stability of SOX9 and Col II increased, and transcriptome analysis showed that genes involved in cell stress responses, cell signaling, and transforming growth factor beta signaling were regulated post-transcriptionally along with altered expression of several microRNAs in articular chondrocytes^32,33^. Therefore, these studies indicate that osmotic pressure in cartilage is closely related to cartilage homeostasis.

In a previous report using kidney cells, both nuclear and cytoplasmic expression of TonEBP increased in response to hypertonicity, suggesting that both cytosolic and nuclear localization of TonEBP protein contributes to its activity as a transcription factor. Interestingly, because considerable portion of TonEBP was found in nucleus under isotonic (basal) conditions without stimuli, its activity can be easily regulated through bidirectional change (increase or decrease) in nuclear fraction of TonEBP in a manner dependent on stimuli^34^. TonEBP is activated by inflammatory cytokines, including TNF-α and IL-1β, as well as osmotic stimuli; osmotic stimuli activate TonEBP by increasing cellular levels and nuclear translocation, whereas inflammatory cytokines induce nuclear translocation of TonEBP without additional TonEBP induction in NP chondrocytes^12^. Increased expression and nuclear translocation of TonEBP were detected in fibroblast-like synoviocytes of RA and OA patients, as well as in TNF-α-, IL-1β-, and NaCl-treated synoviocytes^11^. Furthermore, TonEBP-haplodeficient mice showed decreased arthritis scores in an RA animal model^11^. While TNF-α induced inflammatory responses, but not osmoregulatory genes, in NP cells^12^, our results show that TonEBP protein exists in both the nucleus and cytosol of untreated cells, and cytosolic expression and nuclear localization of TonEBP was more elevated by 29-kDa FN-f, together with induction of osmoregulatory genes. These results suggest that even in chondrocytes, the TonEBP-dependent signaling pathway and its target genes differ according to the type of stimulus and cellular origin.

We previously reported that the damage-associated molecular pattern/TLR-2 pathway is a critical pathway involved in the catabolism and anabolism of cartilage ECM^13,14,18^. Our present results also showed that TonEBP modulated 29-kDa FN-f-induced expression of MMPs via TLR-2. This is consistent with reports that TonEBP signaling is mediated by TLRs. For example, lipopolysaccharide (LPS), an agonist of TLRs, increased TonEBP expression independently of osmolarity both in vivo and in vitro in TonEBP-deficient mice, and its derived macrophages exhibited reduced levels of TLR-2-responsive genes, including inducible nitric oxide synthase 2, IL-6, TNF-α, and Col II^35^. Another study revealed that although LPS and NaCl both use TonEBP as a core transcription factor in RAW 264.7 macrophages, these stimuli mutually inhibited distinct sets of TonEBP targets^36^. Reactive oxygen species were found to function as molecular sensors to discriminate between TLR ligation and osmotic stimuli, directing TonEBP activity toward proinflammatory or hypertonic responses in a context-dependent manner^36^.

Cells recognize altered osmotic pressure via a variety of carriers or channels expressed on cell membranes, subsequently preventing drastic changes in cell volume and damage of cellular components important in the maintenance of cellular function^37^. AQP-1 is an osmolarity-sensitive water channel that is highly expressed in human OA cartilage and in chondrocytes in response to IL-1β^38^. AQP-1 downregulation led to decreased expression of a disintegrin and metalloproteinase with thrombospondin motifs (ADAMTS)-4 in chondrocytes, suggesting a negative influence on cartilage ECM integrity^39^. In rabbit NP cells, AQP-1 expression was reduced by hypo-osmotic pressure, subsequently suppressing rapid swelling^40^. In our study, 29-kDa FN-f increased expression of several calcium channels and AQP-1 as well as organic osmoregulatory genes via the TLR-2/TonEBP signaling pathway, although to a lesser extent compared with the effects of NaCl. It is of note that AQP-1 mRNA expression in response to siTLR2 and siTonEBP differed in control condition. Aquaporin being an important membrane channel involved in the transfer of water and small solutes across cell membrane, we reason that its expression might change sensitively in various context, the functional significance of which is unknown.

The influence of CaN on cartilage ECM homeostasis has been reported; under physiological osmolarity, inhibition of CaN increased expression of Col II and decreased expression of MMPs and ADAMTS-4/5 in OA chondrocytes^31^. A study examining cross-regulation of TonEBP and CaN on cartilage anabolic responses in NP chondrocytes reported that TonEBP positively regulated GlcAT-I promoter activity, while this activity was suppressed by co-expression of CaN, NFAT2, NFAT3, and NFAT4^9^. These results suggest that by controlling ECM synthesis via TonEBP signaling, disc chondrocytes can autoregulate their osmotic environment and accommodate mechanical loading. We revealed that perturbation of intracellular Ca^2+^ distribution affected 29-kDa FN-f-induced MMP expression, and prolonged treatment with 29-kDa FN-f led to increased expression of calcium channels and AQP-1. However, 29-kDa FN-f did not directly induce calcium flow into chondrocytes. Therefore, the CaM/CaN/NFAT signaling pathway, as well as TonEBP, may be involved in 29-kDa FN-f-induced catabolic responses via regulation of intracellular calcium levels.

## Conclusions

In conclusion, we found that 29-kDa FN-f induced nuclear translocation of TonEBP, and that MMP upregulation by 29-kDa FN-f was mediated via TLR-2/TonEBP-dependent signaling. In addition to nuclear translocation of TonEBP, 29-kDa FN-f increased expression of calcium channels and AQP-1 as well as organic osmoregulatory genes via TLR-2/TonEBP signaling pathway. TonEBP may be a promising therapeutic target for managing OA via regulation of the cartilage ECM homeostasis.

## Supplementary Information

Supplementary Information.

## Data Availability

Correspondence and requests for all data required to evaluate the work should be addressed to H.A.K.
