# Supplementary material for: The TLR-2/TonEBP signaling pathway regulates 29-kDa fibronectin fragment-dependent expression of matrix metalloproteinases
Source: Sci Rep. 2021 Apr 26;11:8891. doi: 10.1038/s41598-021-87813-8 (PMC8076285; doi:10.1038/s41598-021-87813-8)
Supplement: Supplementary file 1 — Supplementary Information. [file 41598_2021_87813_MOESM1_ESM.docx]

**The TLR-2/TonEBP signaling pathway regulates 29-kDa fibronectin fragment-dependent expression of matrix metalloproteinases**

Hyun Sook Hwang^a,b#^, Mi Hyun Lee^a,b#^, Hyun Ah Kim^a,b*^

^a^ Division of Rheumatology, Department of Internal Medicine, Hallym University Sacred Heart Hospital, Gyeonggi, 14068, Korea; ^b^ Institute for Skeletal Aging, Hallym University, Chunchon, 24251, Korea

**Table S1. Primer sequences**

| **Gene** | **Primer sequence** |
| --- | --- |
| **TonEBP** | **forward: 5’-CCA-CCA-CCT-GAG-GAC-TTG-CT-3’**  **reverse: 5’-TCC-AAT-CCA-CAC-CCC-TCA-TC-3’** |
| **MMP-1** | **forward: 5’-AGT-GAC-TGG-GAA-ACC-AGA-TGC-TGA-3’**  **reverse: 5’-GCT-CTT-GGC-AAA-TCT-GGC-GTG-TAA-3** |
| **MMP-3** | **forward: 5’-GCG-TGG-ATG-CCG-CAT-ATG-AAG-TTA-3’**  **reverse: 5’-AAA-CCT-AGG-GTG-TGG-ATG-CCT-CTT-3** |
| **MMP-13** | **forward: 5’-AAG-GAC-CCT-GGA-GCA-CTC-ATG-TTT-3’**  **reverse: 5’- TGG-CAT-CAA-GGG-ATA-AGG-AAG-GGT-3** |
| **SMIT** | **forward: 5’-GCA-GAG-CTG-GTT-GCT-CCA-A-3’**  **reverse: 5’-CCA-CAG-GAA-CCA-GCT-TCA-TCA-3** |
| **TauT** | **forward: 5’-CAG-TGG-CAT-CCT-TAG-GTC-ATT-CA-3’**  **reverse: 5’-GCT-GCT-TGC-CCA-TTG-GAG-TA-3** |
| **AR** | **forward: 5’-CAC-TGG-CCG-ACT-GGC-TTT-AA-3’**  **reverse: 5’-GCC-CGA-CTC-ATC-CAA-TGG-3** |
| **VDCC L d type** | **forward: 5’-TTG-GCA-AGC-TGC-AAT-CGA-T-3’**  **reverse: 5’-GGG-TGC-AGA-GGT-GCT-CAT-AGT-T-3** |
| **VDCC L c type** | **forward: 5’-ACG-TCT-TTT-GGA-ATC-AGT-GTC-TTG-3’**  **reverse: 5’-CCG-TAG-GGA-AGC-CCA-ATA-CTT-3** |
| **AQP-1** | **forward: 5’-GCC-TGG-GCA-TCG-AGA-TCA-3’**  **reverse: 5’-TAG-TAG-CCA-GCA-CGC-ATA-GCA-3** |
| **ASIC** | **forward: 5’-CCA-CCGCACCTGCTACCTT-3’**  **reverse: 5’-GGG-CTCCGAGGACACAGA-3** |
| **TRPV4** | **forward: 5’-GGC-TGC-TCC-CAT-TCT-TGC-T-3’**  **reverse: 5’-GAT-GGC-TCT-CGA-AAC-TCC-TCA-T-3** |
| **P2RX7** | **forward: 5’-CGG-TTG-TGT-CCC-GAG-TAT-CC-3’**  **reverse: 5’-** **ACA-ACC-TCG-GTC-AGA-GGA-ACA-3** |

**Table S2. siRNA sequence**

| **Gene** | **siRNA sequence** |
| --- | --- |
| **si-control** | **sense: 5’-CCU-ACG-CCA-CCA-AUU-UCG-U-3’**  **antisense: 5’-ACG-AAA-UUG-GUG-GCG-UAG-G-3’** |
| **si-TonEBP** | **sense: 5’-GAC-CAU-GGU-CCA-AAU-GCA-A-3’**  **antisense: 5’-UUG-CAU-UUG-GAC-CAU-GGU-C-3’** |
| **si-TLR-2** | **sense: 5’-GAU-GUU-AGC-AAC-AAC-AAU-C-3’**  **antisense 5’-GAU-UGU-UGU-UGC-UAA-CAU-C-3’** |
| **si-CaM** | **sense: 5’- CAG-AAC-UUC-GCC-AUG-UGA-U-3’**  **antisense 5’- AUC-ACA-UGG-CGA-AGU-UCU-G-3’** |
| **Si-CaN** | **sense: 5’- CA G-AC A-AU G-GC U-AU A-AA C-U-3’**  **antisense 5’-AGU-UUA-UAG-CCA-UUG-UCU-G-3’** |


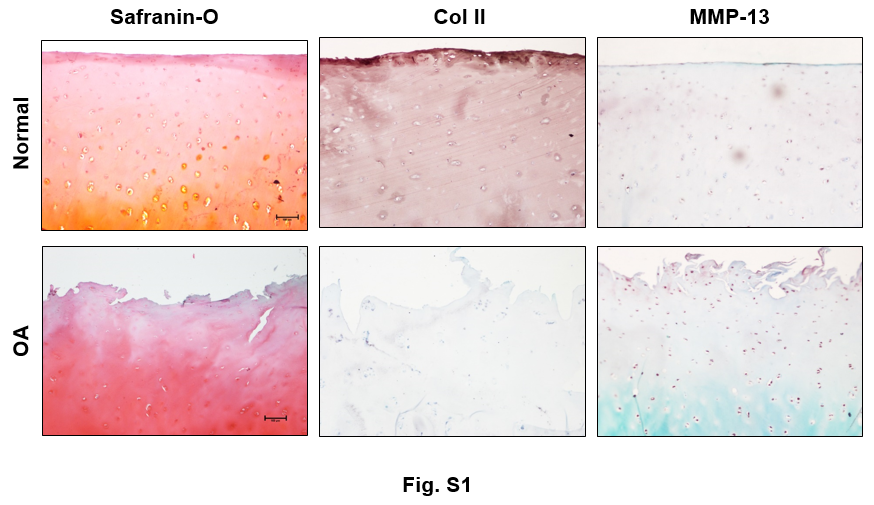
Fig. S1. Safranin O staining and immunohistochemistry of Col II and MMP-13 in normal and OA cartilage.


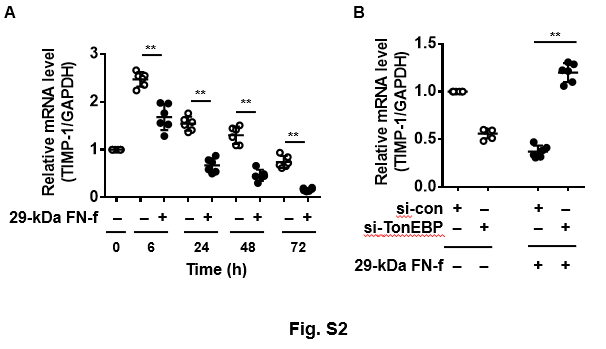


Fig. S2. Effect of 29-kDa FN-f on TIMP-1 level. (A) 29-kDa FN-f suppresses TIMP-1 expression in a time-dependent manner. (B) Change in 29-kDa FN-f-induced suppression of TIMP-1 in TonEBP-knocked down chondrocytes.

**The TLR-2/TonEBP signaling pathway regulates 29-kDa fibronectin fragment-dependent expression of matrix metalloproteinases**

Hyun Sook Hwang^a,b#^, Mi Hyun Lee^a,b#^, Hyun Ah Kim^a,b*^

^a^ Division of Rheumatology, Department of Internal Medicine, Hallym University Sacred Heart Hospital, Gyeonggi, 14068, Korea; ^b^ Institute for Skeletal Aging, Hallym University, Chunchon, 24251, Korea

**Original Blots**


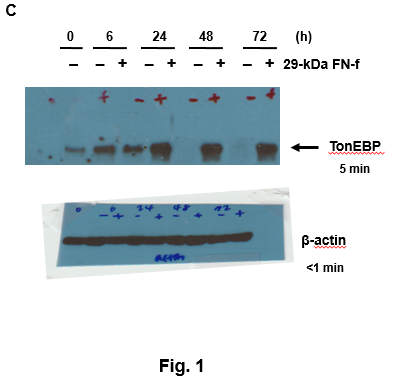


**Supplementary Figure 1.** Display of original blots and exposure time for TonEBP and β-actin Western blot analysis (Fig 1C). Membranes were cut to minimize antibody use. Membranes were separately probed for TonEBP and β-actin.


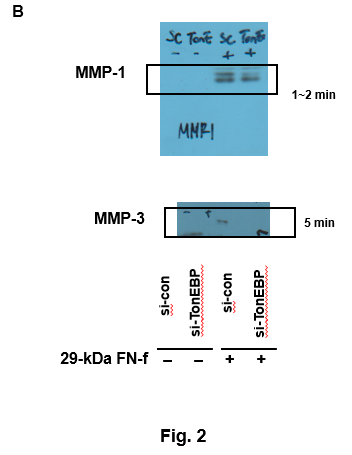


**Supplementary Figure 2.** Display of full length blots and exposure time for MMP-1 and -3 Western blot analysis (Fig 2B). Membranes were cut to minimize antibody use. Membranes were separately probed for MMP-1 and -3.


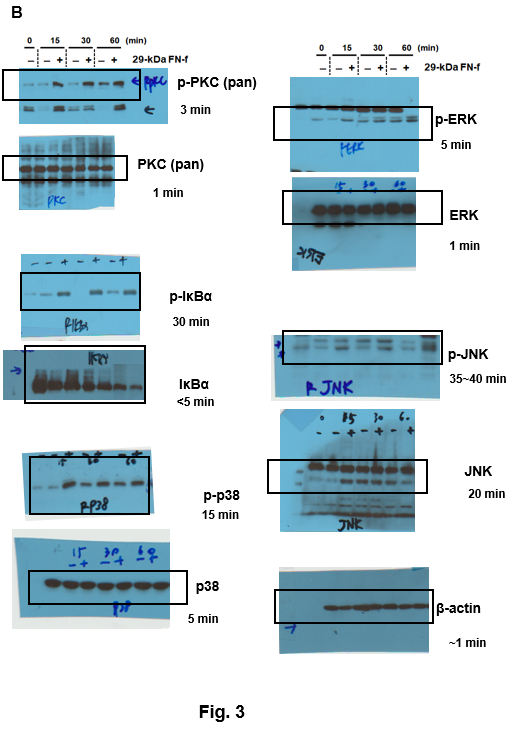

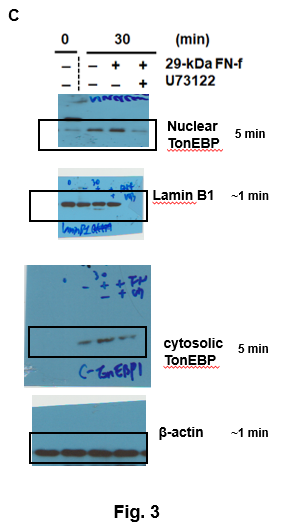

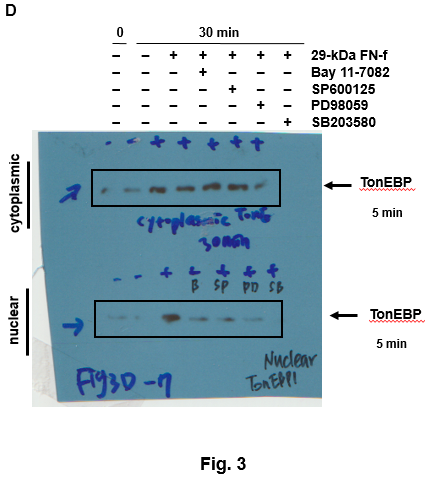


**Supplementary Figure 3.** Display of full length blots and exposure time for (B) p-PKC/PKC, p-Iκ-Bα/Iκ-Bα, p-p38/p38, pERK/ERK, and p-JNK/JNK, (C), (D) nuclear and cytosolic TonEBP Western blot analysis (Fig 3B, C, and D). Membranes were cut to minimize antibody use. Membranes were separately probed for p-PKC/PKC, p-Iκ-Bα/Iκ-Bα, p-p38/p38, pERK/ERK, p-JNK/JNK, and TonEBP .


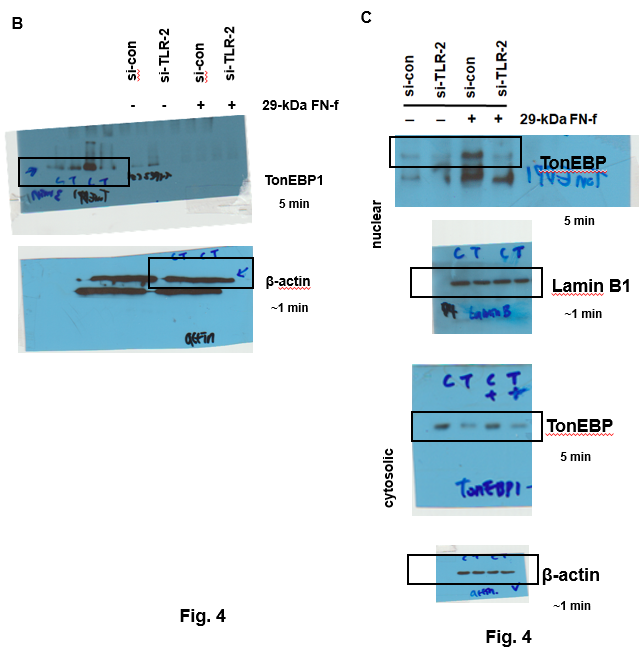


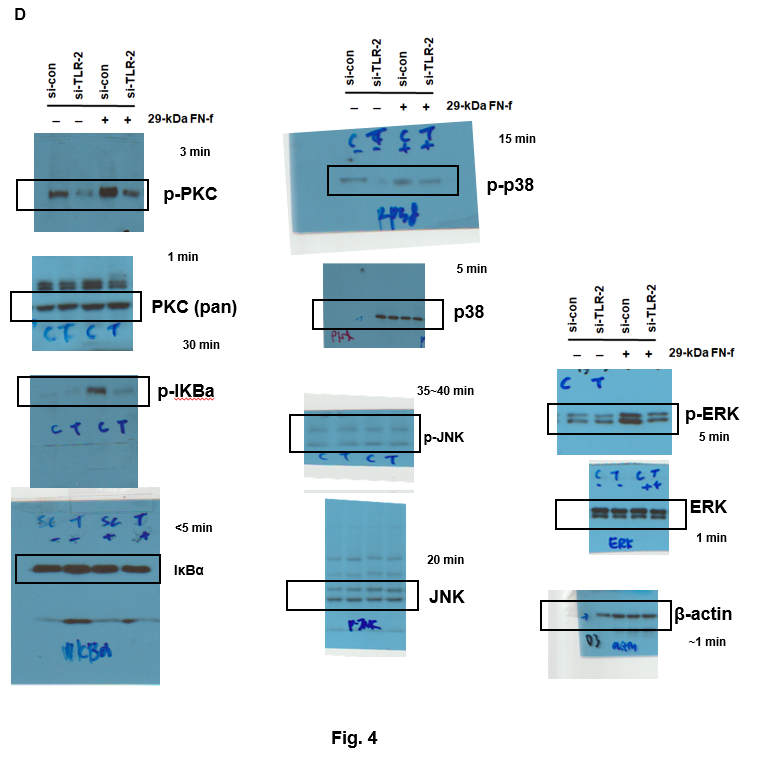


**Supplementary Figure 4.** Display of full length blots and exposure time for (B) TonEBP and β-actin, (C) nuclear and cytosolic TonEBP, and (D) p-PKC/PKC, p-Iκ-Bα/Iκ-Bα, p-p38/p38, pERK/ERK, and p-JNK/JNK Western blot analysis (Fig 4B, C, and D). Membranes were cut to minimize antibody use. Membranes were separately probed for p-PKC/PKC, p-Iκ-Bα/Iκ-Bα, p-p38/p38, pERK/ERK, p-JNK/JNK, and TonEBP.


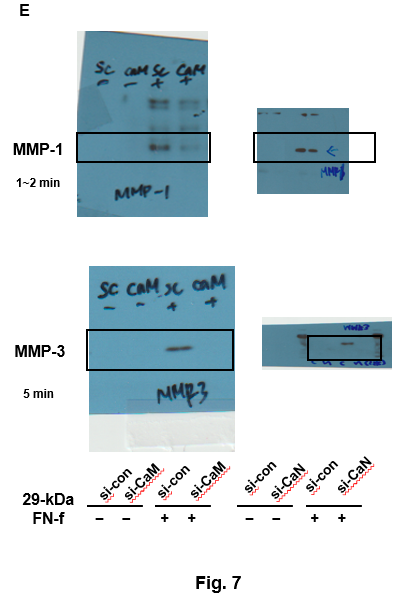


**Supplementary Figure 5.** Display of full length blots and exposure time for MMP-1 and -3 Western blot analysis (Fig 7E). Membranes were cut to minimize antibody use. Membranes were separately probed for MMP-1 and -3.
